# The Effect of Mutations in the TPR and Ankyrin Families of Alpha Solenoid Repeat Proteins

**DOI:** 10.3389/fbinf.2021.696368

**Published:** 2021-07-06

**Authors:** Matylda Anna Izert, Patrycja Emanuela Szybowska, Maria Wiktoria Górna, Matthew Merski

**Affiliations:** Structural Biology Group, Department of Chemistry, Biological and Chemical Research Centre, University of Warsaw, Warsaw, Poland

**Keywords:** protein repeat, Ankyrin repeat, TPR, mutation, sequence function relationship

## Abstract

Protein repeats are short, highly similar peptide motifs that occur several times within a single protein, for example the TPR and Ankyrin repeats. Understanding the role of mutation in these proteins is complicated by the competing facts that 1) the repeats are much more restricted to a set sequence than non-repeat proteins, so mutations should be harmful much more often because there are more residues that are heavily restricted due to the need of the sequence to repeat and 2) the symmetry of the repeats in allows the distribution of functional contributions over a number of residues so that sometimes no specific site is singularly responsible for function (unlike enzymatic active site catalytic residues). To address this issue, we review the effects of mutations in a number of natural repeat proteins from the tetratricopeptide and Ankyrin repeat families. We find that mutations are context dependent. Some mutations are indeed highly disruptive to the function of the protein repeats while mutations in identical positions in other repeats in the same protein have little to no effect on structure or function.

## Introduction

**FIGURE 1:**
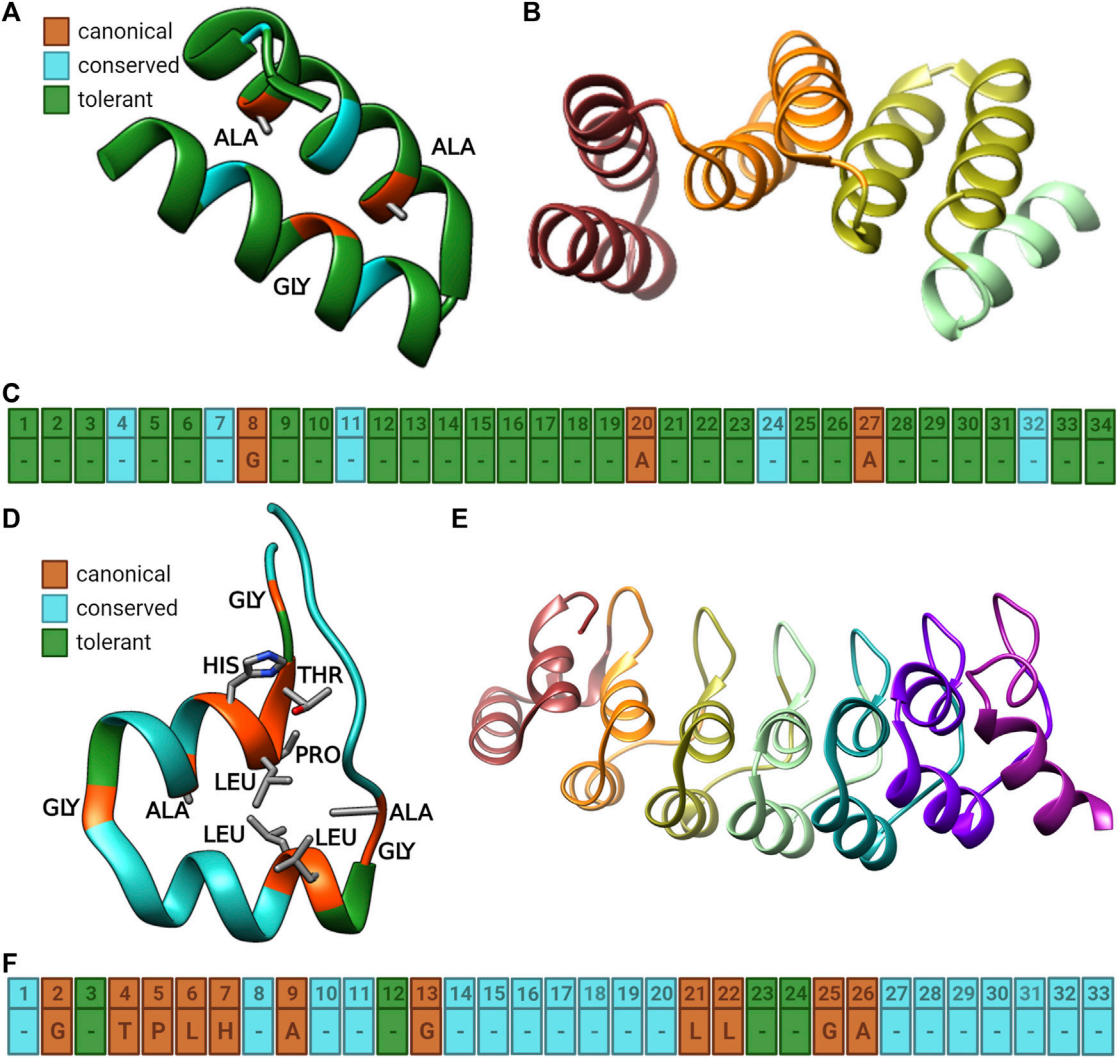
Illustrations of the TPR and Ankyrin repeats (TPR). **(A)** Cartoon diagram of a single TPR repeat showing the canonical (orange), conserved (cyan), and tolerant (green) positions in the repeat using PDB 1NA0. Canonical residues are also shown in stick form with grey carbon, red oxygen, and blue nitrogen atoms. **(B)** Cartoon model of a TPR repeat domain showing four individual TPR repeats, colored by structure to differentiate them using PDB 1ELW. **(C)** Sequence model of the TPR repeat colored as in part A with the canonical residues identified with text (Parra et al., 2015; Kumar and Balbach, 2021). **(D)** Cartoon diagram of a single Ankyrin repeat showing the canonical (orange), conserved (cyan), and tolerant (green) positions in the repeats using PDB 2QYJ. **(E)** Cartoon model of an Ankyrin repeat domain showing seven individual Ankyrin repeats, colored by structure to differentiate them using PDB 4NIK. **(F)** Sequence model of the Ankyrin repeat colored as in part **(A)** with the canonical residues identified with text (Parra et al., 2015; Kumar and Balbach, 2021). Figure was created with BioRender.com and UCSF Chimera (Pettersen et al., 2004).

Protein repeats are stretches of 20–50 residues that repeat (both sequence and structure) within the larger protein (Marcotte et al., 1999; Andrade et al., 2001; Kajava, 2012), with as few as three (Mosavi et al., 2002) to more than 100 occurrences of the repeat with a theoretical limit of 120 repeat units (Galpern et al., 2020). The repeats usually occur sequentially (e.g. tandem repeats) (Kajava, 2012; Jorda et al., 2010), although insertions between repeats can also occur. Sequence repetition in the repeats can be as high as 100% identity (perfect repeats) (Jorda et al., 2010), although lower degrees of similarity (imperfect repeats) are much more common. In theory, any sequence can be the basis of a set of repeats but most identified repeats are restricted to a small set of sequence pattern families (Pallen et al., 2003; Cushing et al., 2005; Champion et al., 2009; Marte et al., 2019). Most of the sequence positions in a repeat are highly variable with only a smaller subset of residues being highly conserved and even the most conserved (canonical) positions display some variability (Stumpp et al., 2003; Kobayashi et al., 2012), although there are often functional costs to deviation at these positions (D’Andrea and Regan, 2003; Severi et al., 2008). However, there tends to be a significant enough restriction of the allowed sequences that the same type of repeat can be identified in distantly related species (Jernigan and Bordenstein, 2014; Schaper et al., 2014; Jernigan and Bordenstein, 2015), even among essentially unrelated species such as bacteria and humans (although lateral gene transfer can never be truly eliminated in these cases) (Schaper et al., 2012). As expected, this sequence restriction results in a high degree of conservation of the secondary structure (Tramontano and Cozzetto, 2005) of the repeats giving rise to highly symmetrical, extended protein structures (Figure 1). As such, local interactions (within a repeat or between neighboring repeats) (Stumpp et al., 2003; Main et al., 2005; Geiger-Schuller et al., 2018) tend to dominate over long range interactions that dominate in non-repeat proteins (Sengupta and Kundu, 2012). As such, repeat sequence repetition may be related to secondary and tertiary structural constraints from these local interactions (Yang et al., 2018) or by co-evolution of interacting surfaces, or some combination of selective pressures (Lovell and Robertson, 2010).

It is possible to classify repeat proteins by their secondary structure composition. For example, the WD40 (Vander Kooi et al., 2010) and Kelch repeats (Severi et al., 2008) are comprised of β-strands arranged into a roughly triangular plane which then collect into a circular shape where the repeats form a topology similar to the blades of a propeller (or the slices of a pizza). Leucine rich repeats contains both a β-strand and an α-helix which in combination stack upon each other to form a solenoid [spiral staircase-like (Magliery and Regan, 2004)] shape, although the strand region is sometimes replaced with a less complex coil structure (Stumpp et al., 2003). Ankyrin repeats (Tripp and Barrick, 2004) also contain α-helices and a conserved ligand binding strand region (Desrosiers and Peng, 2005) and stack into a solenoid structure while the HEAT (Urvoas et al., 2010), Armadillo (Zhao et al., 2009), and tetratricopeptide (TPR) (Grenha et al., 2013) repeats form solenoids from repeats composed of two α-helices. Tetratricopeptide repeats are readily identified by their conserved 34 residue sequence length (Blom et al., 2004) differentiating them from the so-called TPR-like repeats such as the pentatricopeptide (PPR, 35 residues) (Cushing et al., 2005), octotricopeptide (OPR, 38 residues) (Loizeau et al., 2014), and HAT (half a TPR) (Preker and Keller, 1998) repeats. This clustering is more indicative of the structural rather than sequence similarity of these repeats (Paladin et al., 2017; Paladin et al., 2021), although it can be difficult to determine if two classes of repeats are truly separate as there can sometimes be evidence for unexpected relationships [i.e. HEAT and armadillo repeats (Andrade et al., 2001) or LDLreceptorA and LDLreceptorB, or WD40 and PD40 (Turjanski et al., 2016)].

Unfortunately, many of the expected identifying characteristics are variant (or missing) in any specific example repeat. There is no hard rule that fractional repeats cannot occur within a protein, suggesting that the entirety of the repeat does not need to be conserved (Andrade et al., 2001; Pekkala et al., 2004; Clarke et al., 2008; Espada et al., 2015). Their sequences repeat several times making the sequence patterns essentially circular, diluting the meaning of a “starting” or “ending” sequence position (Wall et al., 1995; Marcotte et al., 1999) The identification of the starting residue of a repeat (e.g. the “phase” of the repeat) has been the topic of much discussion (Michaely and Bennett, 1993; Sedgwick and Smerdon, 1999; Mosavi and Peng, 2003; Parra et al., 2013; Parra et al., 2015) and there has been a report of a detectable natural starting pattern for Ankyrin repeats (Parra et al., 2015). Repeat proteins usually, but not always, unfold in a two-state manner despite the fact that repeats can often be freely added or deleted (Tripp and Barrick, 2004; Main et al., 2005; Mello et al., 2005). Nor is repeat length totally conserved as most repeat types have some flexibility in their lengths as well. Increases in the length of TPR repeats, defined by their 34 amino acid length, up to 42 residues have also been reported (Marold et al., 2015). On top of this, the repeating sequences hamper phylogenetic analyses (Andrade et al., 2001; Schaper et al., 2012) of repeat proteins with some repeat regions appearing to change more quickly (Cerveny et al., 2013; Schüler et al., 2016) and others changing more slowly than non-repeat proteins (Schaper et al., 2012). Protein repeat sequences are short enough that they may have arisen more than once in evolutionary history. Functional differences in prokaryotic and eukaryotic repeats point towards this possibility (Marcotte et al., 1999; Kajava, 2001). Further confounding is the observation that in a number of repeat proteins, the functionality is distributed over the full set of repeats, rather than localized to a single repeat (or residue) as is typical for non-repeat proteins (Wang and Lambert, 2010). Which then raises the question, if repeats do not need to be complete to be maintained over evolutionary time and repeat structure (Stumpp et al., 2003), length, and sequence (including the canonical residues) can vary, are mutations in repeats more or less disruptive than in non-repeat regions in light of the fact that the canonical repeat positions are, in fact, highly conserved?

To answer this question, it is necessary to identify what it means for a residue to be conserved. To some extent conservation is what is tolerated by the fold as well as function and the physiology of the host organism (Magliery and Regan, 2004). While simple sequence conservation can be employed (Preker and Keller, 1998; D’Andrea and Regan, 2003), differences in amino acid usage frequencies bias this analysis. Instead, one can also examine the specific effects of mutational substitutions in repeat proteins by comparing changes to the stability and function of the mutant to the naturally occurring proteins. Direct measurements of these parameters allow a quantitative assessment of the impacts of a point mutation. Several models have been developed to analyze these perturbations including one dimensional Ising analysis (Marold et al., 2021), positional frustration analysis (Parra et al., 2015; Espada et al., 2017), use of energy functions like Rosetta (Zhu et al., 2016), and others (Hutton et al., 2015). Free energy analysis can also be used to define sequence conservation, perhaps more accurately than sequence consensus and co-variation analysis has also been applied to both Ankyrin and TPR proteins (Mosavi et al., 2002; Magliery and Regan, 2004) using both real and simulated data to improve the statistical parameters of the analysis (Travers and Fares, 2007). Consensus designs have been shown to generate more stable repeat proteins (Magliery and Regan, 2004), although recent work has identified subtypes within several repeat families suggesting that several notably different consensus designs are possible for a given repeat (Marchi et al., 2019). Care must be taken here however, as the consensus sequence is not necessarily the most stable and residues in contact with ligands tend to be the most variable in repeat proteins (Magliery and Regan, 2004), suggesting a function/stability trade-off (Karanicolas et al., 2011; Houlihan et al., 2015).

In this mini-review, we will examine the role of mutations in repeat proteins using TPR and Ankyrin proteins, two well studied classes of alpha solenoid repeat proteins (Main et al., 2005). Due to the differences between natural and laboratory selective pressures, we will largely avoid designed repeat proteins as well as mutations in capping helices (Main et al., 2003; Stumpp et al., 2003) and mutations that indirectly affect the protein (Boisson et al., 2017). Nor do we claim that this will be an exhaustive list of every mutation ever documented but with enough detail to produce a fairly confident overall assessment of the effects of these mutations. We also note positional numberings are not always uncontroversial for every protein (Lubman et al., 2004; Li et al., 2010). We use the positional numberings from the referenced work or UniProtKB (Bateman et al., 2021).

## Mutations in TPR Proteins

Of the 34 positions in the TPR repeat (Figure 1), sequence conservation identified canonical positions 8, 20, and 27, which are involved in inter helical contacts are the least variable and are typically occupied by alanine or glycine residues (D’Andrea and Regan, 2003; Pallen et al., 2003; Broms et al., 2006; Iakhiaeva et al., 2009; Wang and Lambert, 2010). Residues at conserved positions 4, 7, 11, 24, and 32 are also often restricted to a subset of amino acids, although several of these favor large, aromatic side chains (e.g. positions 4, 11, and 24) and they are also involved in the interaction between the two helices of the repeat. Other positions are more tolerant to substitution; here we use a notation of canonical (the most conserved), conserved (highly conserved) and tolerant as previously established (D’Andrea and Regan, 2003) (Figure 1). This metric has some notable similarity to measurements of protein frustration (Ferreiro et al., 2007) while statistical free energy analysis suggests a slightly different sequence set (Magliery and Regan, 2004). Many TPR proteins have an additional C-terminal helix which does not follow the canonical TPR pattern (D’Andrea and Regan, 2003; Kajander et al., 2009) while others have a divergent N-terminal repeat (Yuzawa et al., 2011). Functionally, TPR domains tend to be protein-protein interaction domains (Blom et al., 2004; Sampathkumar et al., 2008; Wittwer and Dames, 2015; Bidlingmaier et al., 2016) often as an auto-inhibition module (Wu et al., 2001; Yuzawa et al., 2011) but have also been identified as ceramide binders (Bidlingmaier et al., 2016), or involved in chloroplast development (Stanley et al., 2020), γ-secretase activity (Zhang et al., 2012), outer membrane targeting (Koo et al., 2013), and RNA binding (Katibah et al., 2014), among others (Kang et al., 2001). Like many repeat proteins, TPR proteins often have redundant functions (Sampathkumar et al., 2008; Koo et al., 2013). Several TPR proteins have been the targets of significant research interest including protein phosphatase 5 (PP5) (Kang et al., 2001), the C-terminus of hsc70-interacting protein (CHIP) (Wu et al., 2001), and the type III secretion (T3S) system (Broms et al., 2006) which can serve as good models of TPR protein behavior in general.

We will now examine the effects of some mutational changes in TPR proteins (summarized in Table 1). In the *Y. enterocolitica* T3S protein SycD, mutations in several but not all tolerant positions affected binding of one protein ligand but not the other due to their different binding sites, while others and those at conserved positions appeared to affect both (Büttner et al., 2008). Additionally, introduction of charged residues in tolerant positions disrupted dimer formation in some instances but not others (Büttner et al., 2008). In the *P. aeruginosa* T3S protein PcrH, mutations in canonical residues and a double mutant at tolerant positions were found to be greatly detrimental to the protein although all resulted in stable proteins, while mutations in other tolerant and conserved positions did not always destroy the phenotype (Broms et al., 2006). In the *Y. pestis* T3S protein LrcH mutations at tolerant positions conserved, and canonical positions eliminated ligand binding of one or both natural ligands in a yeast two hybrid assay (Edqvist et al., 2006), however, other mutations did not and two mutations in a tolerant positions and several in conserved positions resulted in a negative growth phenotype. Double mutations at the C-terminal end of some repeats allowed crystallization of the peroxin 5 receptor from *T. brucei* by modifying the protein surface to allow the formation of a crystal contact (Sampathkumar et al., 2008). In the nicastrin subunit of human γ-secretase, mutations in tolerant positions were found to be detrimental to activity (Zhang et al., 2012). For the *P. aeruginosa* pilus protein PilF, deletions of repeat 5 or 6 did not reduce phenotypic activity, nor did mutations at a canonical position or several conserved or tolerant positions but mutations at two tolerant and one conserved position did (Koo et al., 2013). In the human RNA binding protein ISG54, mutation of positively charged residues to negative ones at some tolerant positions abolished RNA binding while those at other tolerant positions did not, but did when combined as a double mutant (Yang et al., 2012). In the nucleotide gated channel, TRIP8b, mutations at several (but not all) tolerant positions disrupted the interaction between the TPR domain and the channel (Han et al., 2011). In human SRP72, deletion of repeat 1 or 4 destroyed complex formation (but not protein solubility/stability) while mutations at a set of canonical and conserved positions heavily reduced solubility and also eliminated complex formation although a mutation at one tolerant position also moderately reduced it (Iakhiaeva et al., 2009). In a very thorough report, Kajander et al*.* examined the role of the TPR repeats in Hsp-organizing protein both computationally and experimentally (Kajander et al., 2009). A mutation in a tolerant position in TPR domain 1 greatly reduced affinity for Hsp70, but other tolerant positions had expectedly neutral effects, while in domain 2 several (but not all) tolerant mutations greatly reduced affinity for Hsp90 (Kajander et al., 2009). In collagen prolyl 4-hydroxylase, mutations in four tolerant positions were detrimental to activity while another tolerant mutation was neutral (Pekkala et al., 2004). Finally, in cyclophilin 40, mutations in several tolerant positions eliminated ligand binding while those in other tolerant positions and one conserved position did not (Ward et al., 2002).

**TABLE 1:** Tabular representation of mutational data for TPR. Mutations at a position that notably perturbed the protein function are indicated with an “F” and those that perturbed stability or structure with “S,” and “X” if both function and structure were explicitly reported as affected. Those that were functionally neutral are denoted by “O.” Mutations in positions were specifically noted to have an effect in some instances but not in others are indicated by “#.” The associated literature reference citations are given in grey text under the protein name. At least one mutation was identified for every position except 21 position 4 in the TPRs. A more detailed version of this table is provided as Supplementary Material.

Positon	SycD Büttner et al. (2008)	T3S Broms et al. (2006), Edqvist et al. (2006)	Peroxin 5 recept. Sampathkumar et al. (2008)	FANCG Blom et al. (2004), Wang and Lambert (2010)	γ-secretase Zhang et al. (2012)	PilF Koo et al. (2013)	ISG54 Yang et al. (2012)	O-GlcNAc transferase Rafie et al. (2017)	TRIP8b Han et al. (2011)	SRP72 Iakhiaeva et al. (2009)	HOP Kajander et al. (2009)	Prolyl 4-hydroxylase Pekkala et al. (2004)	Cyclophilin 40 Ward et al. (2002)
1		S								F			
2	X	#				O			F		F		F
3	F	X				F						F	
4	X	X				O							
5	X	X							F		X		F
6	X	X			O	O					#		F
7	#	#		F		#							
8		X		F		O				X			
9		X			#	O	#				X	F	#
10	F	#				F	F						O
11		#				O				X			
12	X	#				O	F				O	F	#
13						O	#		F		#		O
14						O			O				
15									F		O		
16				O		O							
17	O	#				O						F	
18						O						O	
19						O							
20		X		O						X			
21													
22	O					O					F		
23						O							F
24	F	#				O							
25	O					O							O
26	#				O	O							
27		X											
28						O							
29			S		O	O							
30	X		S			O		F					
31	F	O	S			O							F
32	F	#											O
33	#		S					F			S		
34													

While a plethora of mutations have been made in a number of TPR proteins, and changes to some specific residues can result in near complete loss of protein function, many mutations in positions that are highly sequence conserved, (or even elimination of one or more full repeats) can have little or even neutral effects on function, while conversely, some mutations in tolerant positions can be highly detrimental to the function of the TPR protein, although this is expected to be due to loss of specific, localized binding interactions rather than protein unfolding (Table 1). While there was a bit of a bias towards mutational investigation of the N-terminal half of TPR repeats, overall it is clear that context matters for TPR repeats and mutations at canonical positions and even complete deletion of a repeat may have little or no effect on protein function or stability.

## Mutations in Ankyrin Repeats

The Ankyrin repeat is a 33 amino acid repeat consisting of two helices and a ligand-binding loop region (Parra et al., 2015; Kumar and Balbach, 2021). While most of the repeat is fairly well defined, positions 2, 4–7, 9, 13, 21, 22, 25, and 26 are the most conserved while only positions 3, 12, 23, and 24 can be considered tolerant. (Mosavi et al., 2002). (Figure 1, note that there are several numbering systems used for Ankyrin repeats present in the literature (Sedgwick and Smerdon, 1999)). On average, 20% of the residues of an Ankyrin repeat are involved in ligand binding, with the majority (80%) of these being canonical residues and the positions that are most intolerant to mutation tend to be interface interactions between the repeats (Parra et al., 2015). Mutations in Ankyrin repeats are often targeted at positions conserved within a family rather than at the generic repeat structure due to the broader sequence conservation of these repeats compared to TPR proteins (Figure 1) (Tamura et al., 2011). Here we also focus more on mutations in natural proteins rather than designed Ankyrin proteins (Karanicolas et al., 2011) partly because natural proteins are subject to evolutionary pressures that do not affect lab evolved ones although analysis sometimes finds differences between natural and designed repeat sequences (Parra et al., 2015) but not always (Espada et al., 2017). Many designed Ankyrin repeat proteins (DARPins) are generated by random mutation followed by screening so mutations that disrupt protein folding are effectively invisible by virtue of not appearing in the screens (Urvoas et al., 2010; Kummer et al., 2013; Pluckthun, 2015; Schütz et al., 2016).

For integrin-linked kinase, a mutation in a conserved position was detrimental to binding in a pull down assay but one in a tolerant position was not, as expected (Chiswell et al., 2010; Table 2). In the regulatory protein RFXANK, group mutation of the hairpin positions (1, 2, 32, and 33) eliminated glutathione S-transferase binding in the first three but not the fourth repeat, and had no effect on binding to the class II transactivator (Nekrep et al., 2001). Some mutations in conserved positions did not hamper function but mutations in canonical positions in repeat 3 did. Double alanine hairpin loop mutations on any single one of the 24 Ankyrin B repeats did not significantly harm binding to the inositol 1,4,5-trisphosphate receptor, but these mutations in repeat 24 in combination with those in any one of repeats 19–22 did, although the exact positions of these mutations were not fully specified (Mohler et al., 2004). Double mutation of a pair of charged conserved positions to alanine in Ankyrin B abolished its interaction with its own C-terminal membrane binding domain (Abdi et al., 2006). A review of Notch mutations by Lubman et al. (2004) reports that a set of mutations at both canonical and conserved positions as well as a few in the terminal seventh, poorly folded repeat were completely disruptive to function, while other mutations in all three positional classes had a milder effect. In the viral K1 protein, mutations at 20 conserved positions in repeat 2, and both single and combination mutations covering much of the repeat lenght stopped viral replication in HeLa cells (Li et al., 2010). In the VPS9-ankyrin-repeat protein, mutations at several conserved and a tolerant position eliminated activity, but other similar mutations did not (Tamura et al., 2011). Mutations in the human DHHC17 palmitoyl transferase at conserved and tolerant positions prevented ligand binding but two in hairpin positions did not (Verardi et al., 2017). Mutations in the kinesin family protein 21A (KIF21A) AK1 or ANK2 domains in hairpin positions had a moderate (up to 30 fold) effect on binding affinity (as well as some in tolerant and conserved positions) as measured by ITC. These interactions were confirmed by mutation in the peptide ligand (Guo et al., 2018). Simultaneous mutations to alanine at canonical positions of human IκBα always gave soluble protein but did affect complex formation and were most detrimental to activity when in the third repeat (Inoue et al., 1992). Analysis of several cancer-associated mutations in human p16 showed that mutations in all positional three position classes had detectable kinase binding defects and failed to inhibit cell growth (Yarbrough et al., 1999). In gankyrin, almost every mutation tried noticeably affected protein folding by altering a complex folding pathway in which the first three repeats were likely to fold before the four C-terminal repeats. Non-classical Φ-values were also observed for mutations in repeats 1, 2, 3, and 7 in some cases (Hutton et al., 2015). Mutations in the Ankyrin domain of TRPV4 that were known to cause genetic disorders were collected in a book chapter by Kang and were distributed among conserved and tolerant positions (Kang, 2012)

**TABLE 2:** Ankyrin repeats. Mutations at a position that notably perturbed the protein function are indicated with an “F” and those that perturbed stability or structure with “S,” and “X” if both function and structure were explicitly reported as affected. Those that were functionally neutral are denoted by “O.” Mutations in positions were specifically noted to have an effect in some instances but not in others are indicated by “#.” The associated literature reference citations are given in grey text under the protein name. At least one mutation was identified for every position except 21 position 22 in the Ankyrin repeats. A more detailed version of this table is provided as Supplementary Material.

Position	Integrin-linked kinase Chiswell et al. (2010)	RFXANK Nekrep et al. (2001)	Ankyrin B Mohler et al. (2004), Abdi et al. (2006)	Notch Lubman et al. (2004), Del Bianco et al. (2008)	K1 Li et al. (2010)	VPS9 Tamura et al. (2011)	DHHC17 Verardi et al. (2017)	KIF21A Guo et al. (2018)	IκBα Inoue et al. (1992)	p16 Yarbrough et al. (1999)	Gankrin Hutton et al. (2015)	TRPV4 Kang (2012)
1	F	#			F		O	F				
2		#							#			
3					#			F				
4				#	#				#	O		
5				O	#				#	F	S	
6				O					#		S	
7		F		O	#				#			
8		F	F	O	#		F		F	F	S	
9				#						F	#	
10		F		S	F					O	S	
11		F	F	S				F		O		
12	O			#	#	O	F			F		F
13				#	#							
14				#	#	F						
15				O	#	F				O		
16		O		O	#					O		
17				O	F	O	F					
18		O									#	
19		O			#	O						
20					#						#	
21											S	
22												
23		O			F	F						
24		O			F		F					
25							F					
26				X								
27					F		O					
28				X	F		F			F		
29							F					
30				X	F	O						
31					F		O			F		
32		#			F							F
33		#		X				F				F

Ankyrin repeat proteins have more canonically-defined, highly conserved positions than TPR proteins (Figure 1). Curiously, they seem to also exhibit functional delocalization similarly to the TPR repeats (Parra et al., 2015). Functional delocalization is observed when mutations at any single position are compensated by a distributed set of other functional interactions present in the protein [e.g. single point mutations are non-debilitating; in one study 83% of randomly picked in-frame DARPins could be purified as monomers (Seeger et al., 2013) while another found a much weaker sequence restriction in TPR than Ankyrin repeat proteins (Parra et al., 2015; Kumar and Balbach, 2021)]. This delocalization would be in contrast to the majority of functional contribution being generally localized in the catalytic residues in an enzyme active site (Carter and Wells, 1988). For example, it was found that phosphorylation at no single residue was responsible for activity in the yeast Ankyrin repeat protein Pho81p and a greater number of mutations increased activity in some instances (Knight et al., 2004). Distributed weak interactions were also suggested by mutations in Notch when complex formation was eliminated by charge reversing mutations alhough mutation of other charged conserved residues was neutral, as was the removal of a tryptophan (Del Bianco et al., 2008). Additionally, in the TPR-containing Fanconi anemia FANCG protein, mutations at canonical position 8 were detrimental to activity in three repeats and a mutation at position 7 was so in a fourth. (Blom et al., 2004). A mutation at a tolerant position did not harm activity although the binding interaction was suggested to be widely distributed over the entire TPR domain rather than linked to any specific repeat (Wang and Lambert, 2010). Likewise, in human O-GlcNAc transferase, a combined set of mutations at tolerant positions 30 and 33 greatly reduced but did not eliminate activity despite accounting for the bulk of interactions between the TPR domain and substrate (Rafie et al., 2017).

## Conclusion

Repeat proteins tend to be organized by local interactions rather than long distance ones (Main et al., 2005) and positions involved in inter-repeat interactions tend to be more sequence restricted (Parra et al., 2015). Despite the obvious sequence and structural similarities between individual copies of repeats, they can be functionally distinct in that mutations at the same position in different repeats within the same protein are not equivalent. Much like non-repeat proteins, mutations that perturb the structure (the canonical positions which are involved in inter-repeat interactions) are often highly disruptive, but due to the delocalization of structural and functional residues in repeat proteins, many of these mutations do not destroy the function of the protein and it is possible to mutate the canonical residues or even delete entire repeats without disrupting protein function. Studies of mutations in repeats will also be aided by adoption of a standardized positional numbering system of some sort (Han et al., 2011) and we recommend that future reports attempt to do so.
