# Supplementary material for: The Effect of Mutations in the TPR and Ankyrin Families of Alpha Solenoid Repeat Proteins
Source: Front Bioinform. 2021 Jul 6;1:696368. doi: 10.3389/fbinf.2021.696368 (PMC9581033; doi:10.3389/fbinf.2021.696368)
Supplement: Supplementary file 2 [file Table1.pdf]

Table S1: Tabular representation of mutational data for A) TPR and B) Ankyrin repeats. Mutations at a position that notably perturbed the protein function are indicated with an "F" and those that perturbed stability or structure with "S", and "X" if both function and structure were explicitly reported as affected. Those that were functionally neutral are denoted by "O". Mutations in positions were specifically noted to have an effect in some instances but not in others are indicated by "#". Canonical positions are indicated by cyan and conserved positions by grey highlight. At least one mutation was identified for every position except 21 and 34 in the TPRs and position 22 in the Ankyrin repeats.

A)

Positon	SycD	T3S	Peroxin 5 recept.	FANCG	γ-secretase	PilF	ISG54	O-GlcNAc transferase	TRIP8b	SRP72	HOP	prolyl 4-hydroxylase	cyclophilin 40
**1**		S								F			
**2**	X	#				O			F		F		F
**3**	F	X				F						F	
**4**	X	X				O							
**5**	X	X							F		X		F
**6**	X	X			O	O					#		F
**7**	#	#		F		#							
**8**		X		F		O				X			
**9**		X			#	O	#				X	F	#
**10**	F	#				F	F						O
**11**		#				O				X			
**12**	X	#				O	F				O	F	#
**13**						O	#		F		#		O
**14**						O			O				
**15**									F		O		
**16**				O		O							
**17**	O	#				O						F	
**18**						O						O	
**19**						O							
**20**		X		O						X			
**21**													
**22**	O					O					F		
**23**						O							F
**24**	F	#				O							
**25**	O					O							O
**26**	#				O	O							
**27**		X											
**28**						O							
**29**			S		O	O							
**30**	X		S			O		F					
**31**	F	O	S			O							F
**32**	F	#											O
**33**	#		S					F			S		
**34**													

B)

Position	integrin-linked kinase	RFXANK	Ankyrin B	Notch	K1	VPS9	DHHC17	KIF21A	IκBα	p16	Gankrin	TRPV4
**1**	F	#			F		O	F				
**2**		#							#			
**3**					#			F				
**4**				#	#				#	O		
**5**				O	#				#	F	S	
**6**				O					#		S	
**7**		F		O	#				#			
**8**		F	F	O	#		F		F	F	S	
**9**				#						F	#	
**10**		F		S	F					O	S	
**11**		F	F	S				F		O		
**12**	O			#	#	O	F			F		F
**13**				#	#							
**14**				#	#	F						
**15**				O	#	F				O		
**16**		O		O	#					O		
**17**				O	F	O	F					
**18**		O									#	
**19**		O			#	O						
**20**					#						#	
**21**											S	
**22**												
**23**		O			F	F						
**24**		O			F		F					
**25**							F					
**26**				X								
**27**					F		O					
**28**				X	F		F			F		
**29**							F					
**30**				X	F	O						
**31**					F		O			F		
**32**		#			F							F
**33**		#		X				F				F
