# Supplementary material for: The Effect of Mutations in the TPR and Ankyrin Families of Alpha Solenoid Repeat Proteins
Source: Front Bioinform. 2021 Jul 6;1:696368. doi: 10.3389/fbinf.2021.696368 (PMC9581033; doi:10.3389/fbinf.2021.696368)
Supplement: Supplementary file 5 [file DataSheet1.pdf]

Included in the supplementary material for “The Effect of Mutations in the TPR and Ankyrin families of Alpha Solenoid Repeat Proteins“ by Izert *et al.* are the following:

Table 1: A full color version of tables 1 & 2 from the main text

Table 2: A more detailed spreadsheet covering the mutations identified. The protein names are indicated in the first row of each column. For each mutation, the position of the mutation and the initial and final residue identity are included in standard notation, followed by its functional significance and finally the standard position in the repeat of this mutation in parentheses. Mutations that had a noticable functional significance are indicated with an “F”, those that perturbed stability or structure with “S”, or “X” if both function and structure were explicitly reported as affected. Those that were functionally neutral are denoted by “O”.

Table 3: Mutations for the TPR proteins from the spreadsheet in .csv format

Table 4: Mutations for the Ankyrin repeat proteins from the spreadsheet in .csv format
